# Biomedical Applications of Polylactide (PLA) and Its Copolymers

**DOI:** 10.3390/molecules23040980

**Published:** 2018-04-23

**Authors:** Gaetano Giammona, Emanuela Fabiola Craparo

**Affiliations:** Laboratory of Biocompatible Polymers, Department of “Scienze e Tecnologie Biologiche, Chimiche e Farmaceutiche” (STEBICEF), University of Palermo, Via Archirafi, 32 90123 Palermo, Italy

Currently, the interest of the scientific community towards Polylactide (PLA), blended and/or functionalized with other biopolymers, is rapidly increasing due to its great potential as starting material for biomedical applications. This favorable approval arises from its excellent properties, such as biocompatibility, biodegradability, and tunable mechanical and physicochemical properties, as well as the chemical susceptibility that allows to obtain novel polymeric materials. Moreover, PLA and its copolymers can be appropriately processed and engineered to allow a desired biomedical application.

In this special issue, current experimental research focused on several applications of PLA-based materials, such as the production of drug delivery systems, applicable in controlled and/or targeted drug release, and in the fabrication of bioresorbable scaffolds for tissue engineering in regenerative medicine, is reported. 

In their study, Li et al. reported the therapeutic potential of an antimicrobial peptide, KKVVFWVKFK-CONH_2_ (KSL-W), when it is loaded into poly (lactic-co-glycolic) acid (PLGA)/chitosan (CS) composite microspheres as an effective drug delivery system in the treatment of oral infectious diseases such as periodontitis, and also within bone graft substitutes for alveolar bone augmentation, for long-acting bacterial resistance [1].

Craparo et al. reported a study on a PLA-based nanoparticulate carrier margination in relation to RBC concentration (hematocrit) and pressure drop by using a microfluidic system mimicking the hydrodynamic conditions of human microcirculation in vitro [2], highlighting the importance of taking into account RBC–drug carrier interactions and physiological conditions in microcirculation when planning a drug delivery strategy based on systemically administered carriers.

Oledzka et al. designed and proposed a new multifunctional composite based on PLA as a promising bone substitute material for patients with bone tumors or bone metastasis [3]. The material was successfully synthesized starting from hydroxyapatite porous granules doped with selenite ions and bisphosphonate-conjugated biodegradable branched copolymers, obtained by the ring-opening polymerization (ROP) reaction between a hyperbranched bis-MPA polyester-16-hydroxyl initiator, Sn(Oct)_2_, in the presence of l,l-lactide (LLA) and ε-caprolactone (CL).

Most recent advances in tissue engineering in the fields of oral surgery and dentistry have aimed to restore hard and soft tissues. Ceccarelli et al. described the realization and the characterization of two different inorganic scaffolds based on PLGA acid alone or in combination with hydroxyapatite (PLGA/HA-Alos), both in vitro and in vivo, demonstrating that the osteoconductivity of PLGA/HA-Alos and the efficacy of scaffolds in promoting bone-healing in the sinus lift were increased [4]. Krucińska demonstrated the biological properties of low-toxicity PLGA and PLGA/synthetic poly([r,s]-3-hydroxybutyrate) (PHB) fibrous nanocomposite implants for osseous tissue regeneration, by reporting in detail the evaluation of potential biotoxicity and bioactivity [5,6].

Starting from PLGA copolymer, the development of a novel coating solution for surgical sutures, containing the antibacterial substance totarol in combination with PLGA, was described by Reimbold et al. [7]. In particular, it was found that non-absorbable monofilament and multifilament sutures coated with different amounts and ratios of totarol and PLGA were able to inhibit the growth of *Staphylococcus aureus* showing a great potential to reduce the risk of Surgical site infections (SSIs) and post-operative biofilm-formation on suture material, without adverse effects on tissue.

Moreover, PLA also possesses unique properties to be used for the realization of intelligent fabric and/or packaging films releasing particles. Walczac et al. have explored the characteristics of a nonwoven fabric based on the poly(lactide-*co*-glycolide-*co*-trimethylene carbonate) copolymer (PLLAGLTMC), with thermally induced shape memory and a transition temperature around human body temperature, by thermo-mechanical, morphological, and shape memory analyses [8]. In their paper, Li et al., have reported the development of an antimicrobial packaging film from PLA blends incorporating titanium dioxide (TiO_2_) and silver nanoparticles (nano-Ag) by using a solvent volatilization method, which showed favorable properties compared to pure PLA films [9].
